# Implementation and preliminary evaluation of a structured stepwise training program for laparoscopic partial nephrectomy: a single-center pilot study

**DOI:** 10.3389/fmed.2026.1736330

**Published:** 2026-02-03

**Authors:** Yi Zhao, Wenda Wang, Yuzhi Zuo, Zhigang Ji, Xingcheng Wu

**Affiliations:** Department of Urology, Peking Union Medical College Hospital, Peking Union Medical College, Chinese Academy of Medical Sciences, Beijing, China

**Keywords:** laparoscopy, partial nephrectomy, surgical education, teaching and learning, training

## Abstract

**Objective:**

The objective of this pilot study was to assess the effectiveness of a (multimodal) structured training program, using (stepwise) stages of experience, to improve laparoscopic partial nephrectomy (LPN) skills among two groups of urology (URO) trainees. Key metrics of performance (KPIs) were recorded and compared between the groups.

**Results:**

Time to being ready for the first supervised laparoscopic partial nephrectomy (LPN) for the structured training group was significantly less than the control group (5.65 months vs. 6.80 months; *p* < 0.001), as was duration for performing the first LPN (88.15 min vs. 101.35 min; *p* < 0.001). The structured training group received higher faculty performance scores than the control group (8.30 vs. 7.15, *p* < 0.001).

**Conclusion:**

The development of a standardized structured training curriculum resulted in enhanced skill acquisition and an improved initial operative performance compared to a control group. However, while these results are encouraging, they are preliminary and limited by the lack of an equivalent concurrently documented control. Therefore, these results should be interpreted as evidence demonstrating the benefit of a systematic approach to surgical education and require further investigation through multicenter studies.

## 1 Introduction

Renal tumors are one of the most common forms of urinary tract cancer, with an estimated 431,288 cases of kidney cancer (KC) diagnosed globally in 2020 (1). Renal cell carcinoma (RCC) accounts for the majority of renal cancers (90%); this involves renal clear cell carcinoma (70%), renal papillary cell carcinoma (10–15%), and chromophobe renal cell carcinoma (5%) (2). The incidence of renal cancers has continued to rise, primarily due to earlier detection of small renal masses with more advanced imaging. Although renal cancer incidence is increasing, renal cancer-related mortality is stabilizing (3). The classical triad of hematuria, flank pain, and abdominal mass presentation (<15%) is becoming rare, while most early-stage renal cancers are presently diagnosed incidentally (4). It is believed that the risk factors of obesity, hypertension, and smoking account for around 50% of cases of renal cancer (5).

Historically, treatment of renal tumors involved radical nephrectomy (RN) (6); however, in 2010, the European Association of Urology (EAU) guidelines were updated to favor nephron-sparing surgery (NSS), and specifically partial nephrectomy (PN) (7, 8). The primary benefit of NSS is that it prevents loss of renal function while confirming negative margins. PN was originally completed with open surgery, but with advancements in the field, especially with laparoscopic techniques, laparoscopic partial nephrectomy has emerged as a preferred minimally invasive option (9).

While laparoscopic PN offers multiple advantages, it has limitations, including the narrow retroperitoneal space, which limits the positioning of ports, unclear anatomic landmarks, and limitations of laparoscopic instrumentation (10). The advent of the da Vinci robotic surgical system has increased precision (in terms of surgical workflow) by providing better visualization and control of the procedure when needed (11, 12). Added to the advantage of the robotic platform, the use of intra-operative ultrasound, indocyanine green, and novel holographic imaging systems has been shown to reduce the discovery of renal masses from normal renal parenchyma, reduce positive surgical margins, and allow surgical operators to more accurately block the renal artery and ultimately protect renal function (13–15).

Although these advancements exist in surgical practice, the long learning curve of the laparoscopic technique and the lack of formal training for residents in their medical education present barriers to developing laparoscopic skills as junior residents (16). Traditional surgical training has historically relied on the apprenticeship model, characterized by opportunistic, unstructured learning where trainees gain experience through observation and gradual participation in clinical cases without standardized curricula or formal competency assessments (17, 18). While this approach has trained generations of surgeons, it is increasingly recognized as inefficient, highly variable in quality, and potentially inadequate for complex minimally invasive procedures such as LPN (19, 20). This gap limits the opportunity to practice hands-on skills and creates significant barriers to developing the technical proficiency required for advanced laparoscopic surgery.

Given these challenges, there is a clear need to transition from traditional, unstructured apprenticeship models toward formal, structured training curricula that incorporate simulation, deliberate practice, and competency-based progression (21, 22). To address this gap at our institution, we designed and implemented a comprehensive stepwise training model integrating theoretical learning, simulation practice, animal model training, and supervised clinical experience with defined progression criteria at each stage. This study aims to describe this structured training program in detail and report on its initial impact by comparing the training outcomes and early surgical performance of trainees who underwent the structured stepwise program with those of a historical cohort of trainees who were trained via the previous unstructured, opportunistic apprenticeship approach at our institution.

## 2 Materials and methods

### 2.1 Study participants

From April 2020 to April 2024, 40 surgeons who were willing to participate in the study were enrolled. These surgeons were undergoing further training or postgraduate degrees in laparoscopic surgery at our institution. The cohort of 40 surgeons was randomly assigned to the stepwise training group and traditional teaching group according to the random number Table (20 into each). Data relating to demographic baseline information about the two groups were collected, including age, education, title, and previous laparoscopic surgical experience.

**Figure 1:**
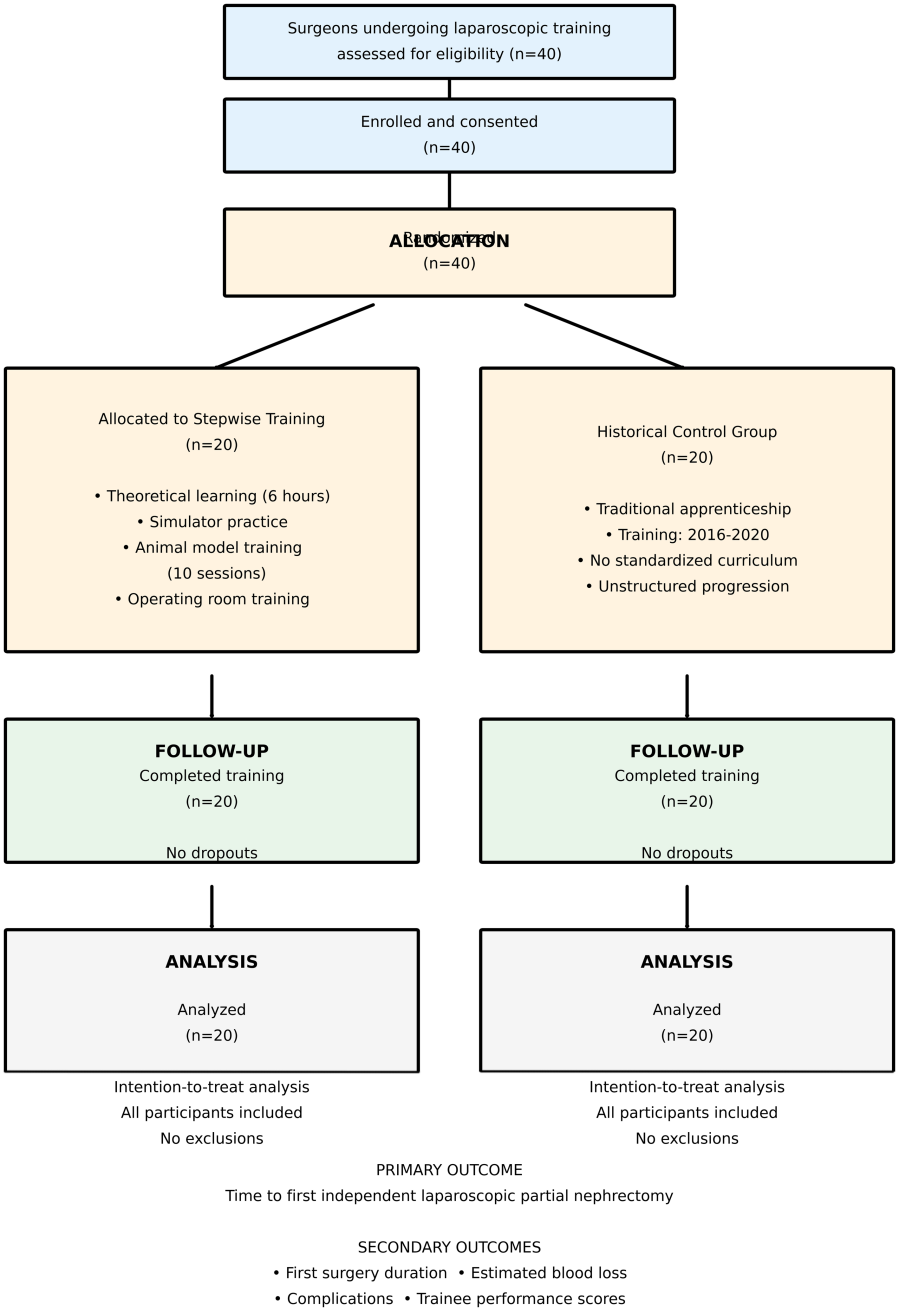
CONSORT flow diagram of study participants. Participant flow diagram showing enrollment, allocation, follow-up, and analysis of trainees in the stepwise training group (*n* = 20, trained 2020–2024) and historical control group (*n* = 20, trained 2016–2020). All 40 enrolled surgeons completed their respective training programs and performed their first independent laparoscopic partial nephrectomy under supervision. No participants were lost to follow-up or excluded from analysis. All outcomes were analyzed using intention-to-treat principles.

The sample size of 40 trainees (20 in each group) was determined based on institutional training capacity and the sample sizes used in other surgical education studies, rather than on formal *a priori* statistical power analysis. However, *post-hoc* power analysis was conducted (see Section 2.9) to assess the study’s ability to detect meaningful differences in training outcomes. A CONSORT-style participant flow diagram is provided in Figure 1.

### 2.2 Training methods

The trainees in the stepwise training group were trained in a structured, stepwise manner consisting of four sequential stages: theoretical study, *in vitro* simulator practice, animal model practice, and operating room practice. Each stage included defined learning objectives and competency-based assessments that trainees were required to pass before progressing to the next stage. The complete stepwise training protocol is described in detail in Sections 2.3–2.6.

The control group consisted of a historical cohort of 20 trainees who underwent training at our institution between April 2016 and April 2020, prior to the implementation of the formal stepwise training program. Their training followed the traditional, unstructured apprenticeship model common in surgical education (17, 18). In this model, there was no fixed learning syllabus or standardized progression criteria. The instructor arranged the contents of lectures, topics of surgical operations, and surgical operation times ‘according to situations’ based on case availability and instructor judgment. Training content and progression were determined by individual instructors without mandated use of simulation or animal models, and detailed logs of training hours and specific activities were not systematically kept at that time.

This historical, unstructured cohort serves as a baseline to evaluate the impact of introducing a structured curriculum at our institution.

### 2.3 Theoretical learning

Complete theoretical learning was guided using instructor-led training time in the topics related to LPN in Urology, Laparoscopic, and Robotic Surgery for a total of 6 h. The theoretical learning topics included indications for LPN surgery, imaging, and pathology of renal tumors, laparoscopic techniques, local kidney anatomy and applied anatomy, complications and complications prevention, and perioperative treatment. At the end of the course, trainees were assessed via a written examination (see Section 2.7), and only trainees who passed the exam were permitted to practice on the *in vitro* simulator.

### 2.4 *In vitro* simulator practice

Trainees used the laparoscopic simulation training system to practice and become acquainted with laparoscopic instrumentation, including intracorporeal imaging systems, separating forceps, needle holders, and scissors. The training included the modular practice of bean-picking, collar positioning, needle-clamping, suturing, and knot-tying. The instructor demonstrated the procedure first, and trainees then practiced under supervision, progressing from easy to more difficult tasks. At the completion of this section, the assessment of the trainee included suturing rubber skin tubes and making a square knot and a slip knot, completing the task in no longer than 10 min. All trainees in the stepwise group successfully passed this assessment before proceeding to animal training.

### 2.5 Animal training

For animal model training, trainees first reviewed a PN surgical video with the instructor. A 25 kg pig was selected as the model. The operation procedure included establishing pneumoperitoneum, Trocar placement, connecting the intracavitary imaging system, and identifying the anatomical landmarks. The trainee was assessed on their ability to successfully skeletonize the renal arteries and veins and perform a PN resection. This training lasted for 10 sessions over 10 days, with 2 h of practice daily. All animal procedures were performed in strict accordance with the ARRIVE 2.0 guidelines and the National Standards for Laboratory Animals of China (GB/T 35892–2018).

#### 2.5.1 Methods of sacrifice

After the 10-day training period, each pig was euthanized by intravenous injection of an overdose of sodium pentobarbital (100 mg/kg) via the auricular vein, followed by bilateral thoracotomy to ensure cardiac arrest and death.

#### 2.5.2 Methods of anesthesia and analgesia

General anesthesia was induced with an intramuscular cocktail of ketamine (15 mg/kg) and xylazine (2 mg/kg). After endotracheal intubation, anesthesia was maintained with 1.5–2.5% isoflurane in 100% oxygen under controlled mechanical ventilation. Intra-operative analgesia was provided by continuous-rate intravenous infusion of fentanyl (5 μg/kg/h) and a single pre-operative dose of meloxicam (0.4 mg/kg IV). Heart rate, oxygen saturation, end-tidal CO2, and rectal temperature were continuously monitored; values remained within physiological ranges throughout all procedures.

#### 2.5.3 Efforts to alleviate suffering

To minimize stress and discomfort, pigs were fasted for 12 h (water ad libitum) and acclimatized to the training facility for 48 h before the first procedure. All surgical fields were infiltrated with 0.25% bupivacaine (2 mg/kg) to provide local analgesia. Post-operatively, pigs received meloxicam (0.2 mg/kg SID IV) for 48 h and buprenorphine (0.02 mg/kg IM q8 h) for 24 h. Animals were housed individually in temperature-controlled pens (22–24 °C) with environmental enrichment and 12 h light/dark cycles; they were monitored at 0, 2, 6, 12, and 24 h after each session for signs of pain or distress using a validated porcine pain scale. Any animal exhibiting sustained pain scores ≥ 3/5 or complications unresponsive to medical management would have been immediately euthanized, although no such cases occurred.

### 2.6 Operating room training

In the operating room, the trainees first observed LPN procedures and then began to participate following a gradual approach. Initial participation involved positioning of the patient, establishing the pneumoperitoneum, and assisting with Trocar placement. Trainees progressed from assisting in camera operation to performing specific procedural steps under direct supervision. As trainees demonstrated competence and gained the confidence of the instructor, they gradually assumed greater responsibility for the procedure. Upon completion of all training stages and demonstration of satisfactory performance across multiple assisted cases, trainees were cleared by the instructor to perform their first independent LPN under supervision. The decision to allow independent performance was based on the instructor’s judgment of overall readiness.

### 2.7 Observation indicators

Theoretical Examination: Both groups received equivalent theoretical examinations to assess their understanding of LPN-related knowledge. This was a written test (score range 0–100), with a passing grade of 80.

#### 2.7.1 Bean-picking assessment

The trainees were required to pick soybeans from one side of a container and place them onto an empty tray within 1 min. The number of soybeans picked within the time limit was recorded.

#### 2.7.2 Suture knotting assessment

The trainees were required to tie 3 effective surgical knots on a suture model within 10 min. The number of effective sutures completed within the time limit was recorded.

#### 2.7.3 Trainee performance score

For the first independent LPN procedure, each trainee’s performance was evaluated by two instructors using a standardized assessment scale (score range 1–10, with 10 being the highest). This score was based on the Objective Structured Assessment of Technical Skills (OSATS) framework, evaluating domains including respect for tissue, time and motion, instrument handling, knowledge of instruments, flow of operation, and overall performance. The final trainee score was calculated as the mean of the two instructors’ ratings.

All skill evaluations were conducted by instructors who were aware of group assignments; blinding of evaluators was not feasible in this educational context.

#### 2.7.4 Study endpoint

The primary endpoint was the ability of trainees to perform their first independent LPN under supervision. Secondary outcome metrics included: surgical training time (months from enrollment to first independent LPN), number of surgical operations observed and assisted before their first independent LPN, first LPN operation time (minutes), estimated blood loss (mL), complications, and trainee performance scores from two instructors.

### 2.8 Statistical methods

The statistical analysis was conducted using SPSS 26.0. Continuous variables were assessed for normality using the Shapiro–Wilk test. For data that were normally distributed, the mean and standard deviation (x̅ ± s) were used, and for data that were not normally distributed, the median and interquartile ranges were used. For data that were normally distributed with equal variance, an independent samples *t*-test was performed, and a non-parametric Mann–Whitney U test was used where distributions differed. Fisher’s exact test was used for categorical variables. A *p*-value < 0.05 was considered statistically significant.

*Post-hoc* power analysis was conducted using G*Power 3.1.9.7 to assess the achieved statistical power for the primary outcomes. For training time (mean difference = 1.15 months, pooled SD = 0.52), the achieved power was >99% (effect size d = 2.21, *α* = 0.05, *n* = 20 per group). For first surgery duration (mean difference = 13.2 min, pooled SD = 8.5), the achieved power was >99% (effect size d = 1.55, *α* = 0.05, *n* = 20 per group). For the complication rate difference (0% vs. 10%, *p* = 0.147), the study was underpowered; approximately 385 subjects per group would be required to achieve 80% power to detect this difference (two-sided Fisher’s exact test, *α* = 0.05).

All analyses were conducted on an intention-to-treat basis. All 40 enrolled trainees completed the study protocol and were included in the final analysis; there were no dropouts or exclusions.

Effect sizes (Cohen’s d for continuous variables, odds ratios for categorical variables) and 95% confidence intervals (CI) were calculated for all primary comparisons to provide estimates of clinical significance beyond statistical significance.

### 2.9 Ethical approval

Approval for this research study was obtained from the ethics committee of Peking Union Medical College Hospital. All human subjects provided written informed consent to participate in this study.

The training using animal models was carried out according to the institution’s guidelines for the ethical use of animals in research. Procedures were reviewed and approved by the Institutional Animal Care and Use Committee of Peking Union Medical College Hospital. All reasonable efforts were made to reduce animal suffering, and all procedures were carried out under the supervision of qualified veterinary staff.

## 3 Results

### 3.1 Baseline characteristics of participants

Before the training, no statistically significant differences existed between the stepwise training group and the historical control group regarding baseline participant information (*p* > 0.05, Table 1). Age, gender, education, professional title, previous laparoscopic surgery experience, and theoretical assessment scores were all comparable between the two cohorts.

**Table 1:** Baseline characteristics of participants.

Characteristic	Stepwise training group (*n* = 20)	Historical control group (*n* = 20)	*t* / χ^2^	*p* value
Age (years), mean ± SD	30.15 ± 3.69	30.95 ± 3.25	0.728	0.471
Gender (male/female), *n*	12/8	13/7	0.107	0.744
Education (Bachelor/Graduate), *n*	6/14	5/15	0.125	0.723
Professional title (Junior/Intermediate/Advanced), *n*	5/12/3	4/13/3	0.151	0.927
Previous laparoscopic surgery experience (Yes/No), *n*	7/13	6/14	0.114	0.736
Theoretical examination score, mean ± SD	75.90 ± 6.32	76.10 ± 6.89	0.096	0.924

SD, standard deviation. *p* values calculated using independent samples *t*-test for continuous variables and Fisher’s exact test for categorical variables. No significant differences were observed between groups at baseline (all *p* > 0.05).

### 3.2 Results of bean-picking assessment

Before training, the two groups did not differ in the number of beans picked (*p* > 0.05). After training, the stepwise training group performed significantly better than the historical control group in the bean-picking assessment (*p* < 0.05, Table 2).

**Table 2:** Bean-picking assessment results.

Time point	Stepwise training group (*n* = 20)	Historical control group (*n* = 20)	*t*	*p* value
Before training	7.45 ± 2.06	7.65 ± 1.95	0.315	0.755
After training	16.85 ± 2.81	11.55 ± 3.27	5.495	< 0.001

Values are mean ± SD number of beans picked in 1 min. *p* values calculated using independent samples *t*-test.

### 3.3 Results of knot tying assessment

Before training, the two groups did not differ in the number of effective sutures completed (*p* > 0.05). After training, the stepwise training group demonstrated significantly better performance in the knot-tying assessment compared to the historical control group (*p* < 0.05, Table 3).

**Table 3:** Suture knot-tying assessment results.

Time point	Stepwise training group (*n* = 20)	Historical control group (*n* = 20)	*t*	*p* value
Before training	2.75 ± 0.72	2.45 ± 0.76	1.285	0.206
After training	11.95 ± 1.99	7.70 ± 1.92	6.877	< 0.001

Values are mean ± SD number of effective surgical knots completed in 10 min. *p* values calculated using independent samples *t*-test.

### 3.4 Comparison of surgical training outcomes

All trainees in both groups completed training and performed their first LPN independently under supervision. Importantly, only patients with T1 renal tumors were selected to ensure patient safety and ethics for the trainees’ first independent procedures. No T2 or larger tumors were assigned. The two groups did not differ significantly in terms of tumor laterality (left vs. right) or maximum diameter of the tumor (*p* > 0.05, Table 4).

**Table 4:** Comparison of training outcomes for first independent laparoscopic partial nephrectomy.

Outcome	Stepwise training group (*n* = 20)	Historical control group (*n* = 20)	*t* / χ^2^	*p* value	95% CI for difference
Patient characteristics					
Tumor laterality (left/right), *n*	9/11	11/9	0.400	0.527	—
Tumor size (cm), mean ± SD	8.05 ± 3.73	7.45 ± 2.89	0.568	0.573	−1.47 to 2.67
Training metrics					
Surgical training time (months), mean ± SD	5.65 ± 0.75	6.80 ± 0.77	4.807	< 0.001	−1.63 to −0.67
Number of training cases, mean ± SD	5.35 ± 0.93	6.55 ± 0.51	5.045	< 0.001	−1.66 to −0.74
First surgery outcomes					
Operation time (minutes), mean ± SD	88.15 ± 8.49	101.35 ± 11.47	4.136	< 0.001	−19.40 to −7.00
Estimated blood loss (mL), mean ± SD	43.95 ± 7.57	47.05 ± 8.20	1.242	0.222	−8.13 to 1.93
Trainee performance score (1–10), mean ± SD	8.30 ± 0.86	7.15 ± 1.04	3.803	< 0.001	0.55 to 1.75
Intraoperative complications, *n* (%)	0 (0.0%)	2 (10.0%)	2.105	0.147	—

SD, standard deviation; CI, confidence interval. *p* values calculated using independent samples *t*-test for continuous variables and Fisher’s exact test for categorical variables. Trainee performance score based on Objective Structured Assessment of Technical Skills (OSATS) framework, rated by two supervising instructors (mean of two ratings). Complications in control group: adrenal bleeding from capsular tears (*n* = 2), managed with absorbable suture and bipolar.

Table 4 compares the training outcomes of the structured stepwise cohort with the historical unstructured cohort. The stepwise training group demonstrated several advantages: trainees achieved readiness for their first independent LPN in significantly less total training time (5.65 ± 0.75 months vs. 6.80 ± 0.77 months, *p* < 0.001) and required fewer training cases (5.35 ± 0.93 vs. 6.55 ± 0.51, *p* < 0.001). Additionally, the duration of the first independent LPN surgery was shorter in the stepwise training group (88.15 ± 8.49 min vs. 101.35 ± 11.47 min, *p* < 0.001). There was no significant difference in intra-operative blood loss between groups (43.95 ± 7.57 mL vs. 47.05 ± 8.20 mL, *p* = 0.222).

Two intra-operative complications occurred in the historical control group (adrenal bleeding from capsular tears, which were managed successfully using absorbable suture and bipolar hemostasis), while no complications occurred in trainees from the stepwise training group during their first independent LPN (0% vs. 10%, *p* = 0.147). The performance scores assigned to the trainees by supervising instructors were significantly higher in the stepwise training group (8.30 ± 0.86 vs. 7.15 ± 1.04, *p* < 0.001, Table 4).

## 4 Discussion

The primary finding of this pilot study is that the implementation of a structured, multimodal training curriculum was associated with significantly accelerated skill acquisition compared to our institution’s previous unstructured, apprenticeship-based approach (23). Our data indicate that trainees in the stepwise group not only achieved proficiency faster but also demonstrated superior intraoperative performance, as evidenced by higher instructor-assessed scores and a complete absence of intraoperative complications. This suggests that a systematic, phased approach to surgical education can yield more efficient and safer learning curves for complex procedures like laparoscopic partial nephrectomy (LPN) (24).

### 4.1 Limitations

The primary limitation of this study is the use of a historical, non-contemporaneous control group whose training was not prospectively documented with the same rigor as the intervention group. This design prevents a direct, fair comparison between two well-defined pedagogical methods and introduces a significant risk of confounding variables. Specifically, temporal changes in technology, equipment, or institutional practices may have occurred between the control period (2016–2020) and the intervention period (2020–2024), and there may have been potential differences in faculty experience or teaching approaches over time. Additionally, the lack of detailed documentation of the control group’s actual training activities, hours, and progression criteria, combined with the inability to control for total training volume or resource allocation differences between groups, further compromises the validity of direct comparison.

Therefore, the results of this study cannot be used to conclude the superiority of the stepwise training model over other structured training methods or over a well-documented traditional curriculum. Instead, the findings should be interpreted as a preliminary evaluation of the impact of implementing a formal, structured, competency-based curriculum at our institution compared to the previous unstructured, opportunistic apprenticeship approach.

Additional limitations merit discussion. The lack of blinding represents a significant concern, as instructors who evaluated trainee performance were aware of group assignments, introducing potential observer bias. As a single-center study, the findings may not be generalizable to other institutions with different resources, patient populations, or training cultures. The small sample size employed in this study was underpowered to detect differences in rare outcomes such as complications. Furthermore, trainee competency was assessed based on their first independent laparoscopic partial nephrectomy; this assessment does not necessarily reflect sustained competence or performance on subsequent cases. The study also lacked long-term follow-up, preventing any assessment of skill retention, performance on subsequent cases, or long-term clinical outcomes. Finally, important metrics such as warm ischemia time, positive surgical margin rates, and postoperative renal function were not systematically collected, representing missing key surgical outcomes.

The principal value of these findings, while preliminary, lies in the profound importance of structure itself in surgical education. The observed gains are likely attributable to the core tenets of a structured curriculum: defined milestones, deliberate practice, and integrated simulation (25). The traditional apprenticeship model, while historically significant, often lacks standardized evaluation metrics and relies heavily on chance case exposure, a weakness that has been widely critiqued (26). In contrast, our four-phase model ensures that every trainee masters foundational knowledge and basic psychomotor skills before advancing to more complex tasks. The theoretical learning phase established a uniform cognitive baseline, while the simulator and animal model phases provided a safe, low-stakes environment for deliberate practice, allowing for repetition and refinement of technical skills without compromising patient safety. This progressive increase in complexity and fidelity is a hallmark of competency-based medical education and appears to be the primary driver of the accelerated skill acquisition we observed (27).

The most significant limitation of this study, which must be emphasized, is the nature of the control group and the non-randomized design. This is not a direct comparison of two distinct educational methods, but rather an evaluation of a structured intervention against an undocumented, historical baseline. The “traditional training group” represents a retrospective cohort whose training was based on the classic apprenticeship model, which by its nature is heterogeneous and lacks standardized documentation. Consequently, we cannot control for confounding variables such as instructor variability, case diversity, or individual trainee aptitude between the two groups. This fundamental flaw, often cited as a major challenge in educational research (28), means that while our results are encouraging, they must be interpreted as a strong signal of the potential benefits of structured training rather than definitive proof of the superiority of our specific model over another.

Our findings align with a growing body of literature advocating for a paradigm shift away from the traditional Halstedian “see one, do one, teach one” model toward competency-based medical education (29). The weaknesses of the apprenticeship model, including its variable quality and potential risks to patient safety, are well-documented (26). Conversely, simulation has been repeatedly shown to reduce the clinical learning curve and decrease adverse events (24). High-fidelity simulation, in particular, improves adaptability and readiness for real-world surgical challenges (25). By integrating theoretical learning, low- and high-fidelity simulation (animal models), and supervised clinical practice, our curriculum embodies the principles of structured, competency-based training that the literature increasingly supports as a best practice for surgical education (30).

Beyond the critical issue of the control group, several other limitations must be acknowledged. First, as a single-center study with a relatively small sample size, the generalizability of our findings is limited. The results may not be directly applicable to institutions with different resources, patient populations, or baseline educational standards. Second, the lack of blinding for instructors and evaluators introduces a significant risk of observer bias, a well-known factor that can influence subjective assessments in clinical studies (31–34). Although structured evaluation criteria were used, the knowledge of a trainee’s group assignment could have subconsciously influenced scoring. Future studies must incorporate blinded assessment to validate these performance metrics. Third, the study lacks long-term follow-up, preventing any assessment of skill retention or the transferability of laparoscopic partial nephrectomy skills to other complex procedures. Finally, the study was ethically restricted to T1 tumors, which precludes the evaluation of trainee performance in higher-complexity cases. Future research should aim to address these limitations through multicenter, randomized controlled trials with blinded evaluation and long-term follow-up, which are considered the gold standard for validating the efficacy of educational interventions (32).

## 5 Conclusion

The implementation of a structured, stepwise training program was associated with faster skill acquisition for LPNs in this single-center pilot study when compared to a historical, unstructured training cohort. However, due to fundamental limitations in the study design, most notably the lack of a prospectively documented control group, these findings should be considered preliminary and hypothesis-generating. The results do not prove the superiority of this specific stepwise model, but rather highlight the potential value of implementing any structured, competency-based curriculum over traditional, opportunistic learning. Rigorous, multicenter randomized controlled trials comparing different, well-defined training curricula are urgently needed to establish best practices in surgical education.

## Data Availability

The raw data supporting the conclusions of this article will be made available by the authors, without undue reservation.
